# Clinician-Reported Consequences and Patient Outcomes in Disputed Emergency Department Admission Referrals: A Systematic Review

**DOI:** 10.7759/cureus.118702

**Published:** 2026-10-28

**Authors:** Laiba Kayani, Hafsah Azam, Ayman Emam

**Affiliations:** 1 Trauma and Orthopaedics, King's Mill Hospital, Sutton-in-Ashfield, GBR; 2 Medical School, Medical University of Plovdiv, Plovdiv, BGR; 3 Cardiology, Nottingham City Hospital, Nottingham, GBR

**Keywords:** emergency department, emergency department boarding, hospital admission, interprofessional conflict, patient handoff, patient safety, referral and consultation, systematic review, turfing

## Abstract

Patients in the emergency department (ED) who need admission depend on an inpatient team agreeing to take responsibility for their care. This systematic review examined patient outcomes, patient experiences, and clinician-reported consequences associated with inpatient specialties disputing, declining, redirecting, or deferring ED admission referrals. The review was prospectively registered on PROSPERO (CRD420261538117) and reported according to the Preferred Reporting Items for Systematic Reviews and Meta-Analyses (PRISMA) 2020 statement. MEDLINE, Embase, Cumulative Index to Nursing and Allied Health Literature (CINAHL), and the Health Management Information Consortium database were searched on 12 October 2026, and the reference lists and citing articles of included studies were checked. Two reviewers independently screened records, extracted data, and appraised quality using the Mixed Methods Appraisal Tool; findings were synthesised narratively, and confidence in qualitative findings was assessed with GRADE-CERQual. Twenty-four studies reported in 28 articles (2005-2026) from 10 countries were included, five of them identified by citation searching. Most of the evidence came from clinicians’ reports, and only one study reported outcomes for patients known to have had a disputed referral: in that audit of 109 rejected admission requests, 97 patients (89.0%) were eventually admitted, 66 (60.6%) under the first team contacted, and ED length of stay increased with the number of teams involved. Two record-based studies counted disputes or refusals without reporting outcomes separately for the patients affected. Two hospital-wide interventions that limited bed rejections or clarified emergency physician admitting authority were followed by shorter waits, without concurrent controls. In one interview study, patients who were receiving residents considered inappropriately transferred described more negative experiences. In a qualitative analysis of survey accounts, 40 of 139 physicians (29%) reported that a patient had experienced an adverse event or near miss after transfer from the ED; in a separate survey, 36 of 126 physicians (29%) reported a handoff-related adverse event or near miss. Clinicians attributed disputes to diagnostic uncertainty, workload, bed pressure, unclear or unequal rules on which team must accept, referral timing, and specialty identity and hierarchy. Disputes over which team accepts an ED admission are commonly described, rarely measured, and a potentially modifiable source of delay, but their effect on patient outcomes cannot yet be estimated. Recording the number of teams contacted and the time from first referral to acceptance would allow hospitals to measure the problem and test solutions.

## Introduction and background

The emergency department (ED) depends on inpatient specialties to accept patients who need admission. When that acceptance is slow, the consequences are well-described: crowding, prolonged boarding, and delayed care are associated with worse outcomes across health systems [1]. In England, 30-day mortality rose when the time from ED arrival to an inpatient bed exceeded five hours, with one extra death for every 82 admitted patients delayed by six to eight hours [2]. In a United States cohort of 41,256 admissions, in-hospital mortality rose from 2.5% in patients boarded for less than two hours to 4.5% in those boarded for 12 hours or more [3].

Much of this delay is attributed to bed capacity [1]; yet some begins earlier, during the conversation about which team should accept the patient. An inpatient specialty may decline a referral, request additional investigations, redirect the patient, or defer acceptance pending senior review. Clinicians often call the transfer of an unwanted patient between teams "turfing," a term that implies avoidance of responsibility rather than a considered decision about where the patient belongs [4]. Disagreement between professionals is not inherently harmful and can improve decisions; however, unresolved disagreement may leave a patient without a clearly responsible team [5].

Previous systematic reviews have examined ED crowding [1], the association between boarding and in-hospital mortality [6], consultation in the ED [7], failures of handoff communication during transfers within hospitals [8], and interventions to improve ED patient flow [9]. These reviews treat the wait for an inpatient bed as one interval, or focus on how often consultations occur and what information is lost at transfer; none examined what happens to patients when the inpatient team disputes, declines, or defers the referral. Before registering this review, we checked PROSPERO for similar registered reviews and found none, and no systematic review of this question was identified among the records screened. Because disputes are rarely recorded in routine data, we expected that much of the evidence would come from clinicians’ accounts rather than measured patient outcomes, and the review was designed to include both.

This systematic review therefore asked: in adults referred from the ED for hospital admission, what patient outcomes and experiences are associated with inpatient specialties disputing, declining, redirecting, or delaying acceptance of the referral? Secondary aims were to describe the contributing factors and mitigating strategies reported by clinicians.

## Review

Methods

Protocol and Registration

The protocol was registered on PROSPERO (CRD420261538117) on 12 October 2026, before screening began. The review was conducted and is reported according to the Preferred Reporting Items for Systematic Reviews and Meta-Analyses (PRISMA) 2020 statement [10]; a completed PRISMA 2020 checklist is provided in the Appendices (Table 11). Database searches were run on 12 October 2026; title and abstract screening was completed on 13 October; full-text screening on 15 October; and data extraction and quality appraisal on 15 October 2026. Citation searches were run on 23 October 2026, and screening, data extraction, and appraisal of the records they identified were completed on 23 October 2026. Changes from the protocol are described at the end of this section.

Eligibility Criteria

Studies were eligible if they included adults in the ED who were referred to an inpatient specialty for admission, or clinicians who reported on such referrals. The exposure was an inpatient specialty disputing, declining, redirecting, or delaying acceptance of an ED admission referral, so that responsibility for the admission was contested or unassigned for a period. This included "turfing," refusal of referral, admission negotiation, and patients passed between specialties before admission. Policies or interventions aimed at reducing such disputes were also eligible. Studies of general team conflict without a dispute over admission, nurse-doctor conflict, ward transfers after admission, transfers between hospitals, and intensive care unit triage decisions based only on bed availability were excluded, as were studies of children only, primary care, and patients referred only to mental health or maternity services.

A study was judged to report a dispute over admission if it described an inpatient team declining, disputing, redirecting, or delaying acceptance of an ED patient referred for admission, or a disagreement between ED and inpatient clinicians about whether, where, or under which team the patient should be admitted. Transfer to another hospital was eligible only when it was reported as a consequence of such a refusal by the hospital’s own inpatient team, not as the exposure. Disputes over admission are often reported within broader studies of ED crowding, handover, consultation or clinician wellbeing. Such studies were eligible only if they reported data meeting this definition, and only those data were extracted. Studies in which conflict, handover failure or crowding was described without reference to acceptance of an admission referral were excluded.

The main outcomes were time from referral to acceptance, ED length of stay, ED boarding or 12-hour waits, adverse events, admission to an inappropriate specialty, hospital length of stay, and mortality. Additional outcomes were patient-reported experience and clinician-reported consequences for patients, contributing factors, and mitigating strategies. Any empirical primary study design was eligible. Editorials, commentaries, opinion pieces, ethics discussions, case reports, conference abstracts, preprints, trial registry records, and reviews were excluded. Only English-language publications were included, with no date restriction. Where more than one report described the same participants, the reports were linked and counted as one study, and data relevant to the review were taken from any of the linked reports.

Information Sources and Search Strategy

Ovid MEDLINE (1946 to 9 October 2026), Ovid Embase (1974 to 8 October 2026), CINAHL (EBSCOhost), and the Health Management Information Consortium database (Ovid, 1979 to August 2026) were searched on 12 October 2026 through National Health Service institutional access. The searches combined free-text terms and subject headings for disputed, refused, or deferred responsibility and interspecialty conflict with terms for the ED. Responsibility terms included turfing or turfed, ownership of patient care, most responsible physician, responsibility near disputed, unclear or shifted, referral or admission near refused, declined, rejected or negotiated, and conflict or disagreement near handover, admission or disposition. Subject headings included Interprofessional Relations and Referral and Consultation in MEDLINE and CINAHL, and interdisciplinary communication and patient referral in Embase. ED terms included emergency department, emergency room, emergency medicine, accident and emergency, and the corresponding subject headings. The specific term "turfing" was also searched without the setting limit in CINAHL and the Health Management Information Consortium database. Results were limited to English where the database allowed. In Embase, conference abstracts were removed within the search, and preprints and trial registry records were removed before screening. Two known relevant studies were used to test the strategy and were retrieved by the MEDLINE, Embase, and CINAHL searches. The full search strategies, with the number of records retrieved at each line, are provided in the Appendices (Tables 5-8).

Because "turfing" and related terms are colloquial and inconsistently indexed, the database searches were supplemented by citation searching. On 23 October 2026, the reference lists of all 21 reports included from the database searches were checked (backward citation searching), and articles citing each of these reports were identified (forward citation searching) in Scopus for 18 reports and in Google Scholar for the other three. Citation searching was a single round: the reference lists and citing articles of reports identified by citation searching were not checked.

Study Selection

Records were deduplicated and screened in Rayyan (Rayyan Systems Inc., Cambridge, USA) with blinding switched on [11]. Two reviewers (LK and HA) independently screened all titles and abstracts, and any record either reviewer considered potentially eligible proceeded to full-text review. The same two reviewers independently assessed full texts against the eligibility criteria and recorded one main reason for each exclusion, using the categories wrong exposure, wrong population, wrong outcome, wrong publication type, and, for citation-search reports, not in English. Disagreements were resolved by discussion, and a third reviewer (AE) adjudicated where agreement could not be reached. Agreement was measured with percentage agreement and Cohen’s kappa, interpreted using the categories of Landis and Koch [12]. Reports excluded at full text are listed with reasons in the Appendices (Table 10).

Records from citation searching were deduplicated against each other and against reports already included or assessed in full. When the citation records were added to the review in Rayyan, 263 of the remaining records were identified as having also been retrieved by the database searches; they were not removed and were screened again. LK and HA screened the titles and abstracts of all these records independently, using the same criteria and the same rule for proceeding to full text. LK and HA each checked every criterion against the full text independently and recorded their own decision, and AE resolved disagreements.

Data Extraction

Data were extracted using a form piloted on two included studies. Items were author, year, country, setting, design, participants, sample size, referring and inpatient services, type of dispute, how the dispute was defined or measured, the main and additional outcomes, contributing factors, mitigating strategies, key findings, author-stated limitations, and funding. Two reviewers (LK and HA) extracted data from all included studies independently, each blind to the other’s entries, and a third reviewer (AE) checked every item against the full text.

Risk of Bias and Quality Appraisal

After data extraction, the same two reviewers independently appraised each study, blind to each other’s ratings, with the Mixed Methods Appraisal Tool (MMAT) version 2018 [13]. The MMAT provides two screening questions and five design-specific criteria for qualitative, randomised, non-randomised, quantitative descriptive, and mixed-methods studies. The criteria address, for example, the coherence of qualitative data collection and interpretation, the representativeness of samples, the risk of nonresponse bias, and confounding in non-randomised comparisons. Disagreements were resolved by discussion, with AE adjudicating. In line with MMAT guidance, no overall score was calculated, and appraisal informed interpretation rather than inclusion.

Synthesis

Meta-analysis was not planned because designs, exposures and outcomes were expected to differ substantially. Measured patient outcomes were grouped into five domains, namely, delay (time to acceptance, ED length of stay and boarding), disposition (eventual admission, admitting team and transfer), hospital length of stay, adverse events, and mortality, and were tabulated by study following Synthesis Without Meta-analysis (SWiM) guidance [14]. For each study, we recorded whether outcomes were reported separately for patients with a disputed referral. No effect measures were pooled; outcomes are presented as reported by each study. Where a study reported a proportion with its numerator and denominator, we calculated a 95% confidence interval (CI) with the Wilson score method; other CIs are given as reported. Differences between studies were explored descriptively by design, setting and data source, and no sensitivity analyses were performed. Risk of reporting bias across studies was not formally assessed because no meta-analysis was conducted.

Patient-reported experience and clinician-reported consequences, contributing factors and mitigating strategies were synthesised using thematic synthesis, in which line-by-line coding generated descriptive themes that were then developed into analytical themes [15]. Studies of interventions intended to reduce disputes were summarised separately. Confidence in each finding from the thematic synthesis was assessed with the GRADE-CERQual approach [16], considering methodological limitations, coherence, adequacy of data and relevance, and rated as high, moderate, low, or very low. LK and HA made these judgements independently, and disagreements were resolved by discussion with AE; they reassessed each finding to which a study identified by citation searching contributed. Measured outcomes were not graded with GRADE because no study compared patients with and without a disputed referral; their strength was judged from study design and MMAT appraisal.

Deviations From the Protocol

Citation searching of the reference lists of included studies, which the protocol specified, was completed after peer review rather than before submission. Forward citation searching (in Scopus and Google Scholar for three reports), the GRADE-CERQual assessment, full-text extraction and appraisal for citation-search reports, and exclusion of citation-search reports whose full text was not in English were added at that stage. The reference lists of relevant reviews, also specified in the protocol, were not checked. These changes have been recorded on PROSPERO (CRD420261538117).

Data Availability

The screening records, data extraction forms and quality appraisal forms are available from the corresponding author on reasonable request.

Results

Study Selection

The database searches identified 1,995 records: 614 from MEDLINE, 918 from Embase, 445 from CINAHL, and 18 from the Health Management Information Consortium database. Before screening, 701 duplicates, six preprints, and 99 trial registry records were removed, leaving 1,189 records for title and abstract screening (Figure 1). Of these, 1,147 were excluded. Agreement between reviewers was 92.0% (κ = 0.41, moderate). Forty-two reports were sought for retrieval, and one could not be obtained. Of the 41 reports assessed in full, 20 were excluded: 11 for the wrong exposure, six for the wrong publication type, two for the wrong population, and one for the wrong outcome (Table 10 in the Appendices). Full-text agreement was 80.5% (κ = 0.55, moderate), and the third reviewer adjudicated seven decisions. The remaining 21 reports described 19 studies [17-37], because two studies were each described in two reports: the two reports by Chan et al. described the same 61 physicians at the same four hospitals [32,33], and the journal article by Sujan et al. reported findings from the same 270 observed handovers and 39 interviews as the full project report of that study [35,37]. The project report was retrieved by the database searches and was initially counted as an exclusion because its handover findings were not specifically linked to admission disputes; upon checking for linked reports, it was reclassified as a second report of the included study. For the same reason, data on admission disputes for this study came from the journal article [35].

**Figure 1:**
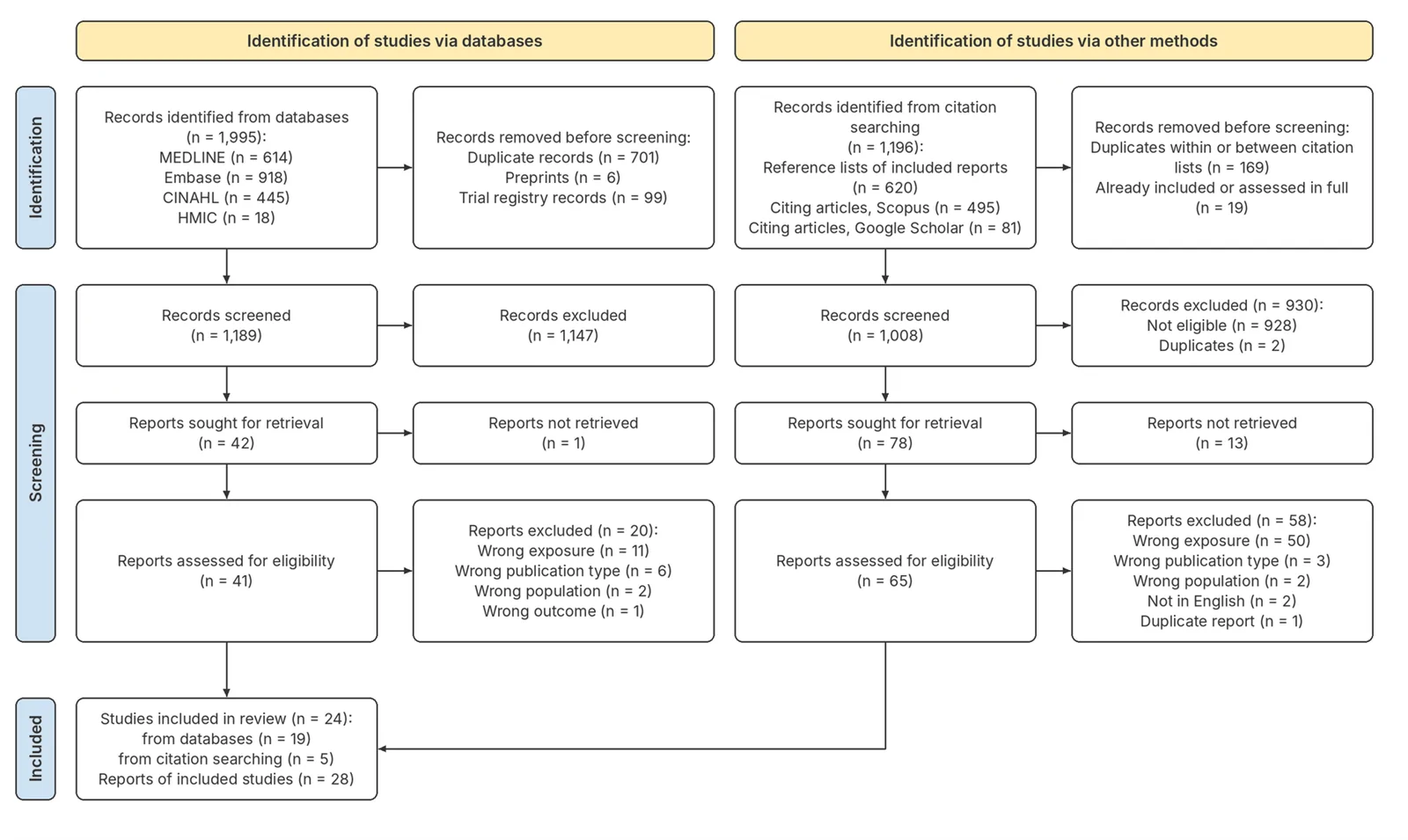
PRISMA 2020 flow diagram of study selection PRISMA: Preferred Reporting Items for Systematic Reviews and Meta-Analyses; HMIC: Health Management Information Consortium The database searches contributed 21 reports of 19 studies, and citation searching contributed seven reports of five studies; two studies were each described in two reports and one in three reports [32,33,35,37,40-42]. Records from citation searching that were already included or assessed in full were removed before screening; 263 records that had also been retrieved by the database searches were screened again. Adapted from the PRISMA 2020 flow diagram template [10]

Citation searching identified 1,196 records: 620 references from the 21 included reports and 576 citing articles (495 from Scopus and 81 from Google Scholar). Before screening, 169 duplicates within or between these lists and 19 records already included or assessed in full were removed, leaving 1,008 records, 263 of which had also been retrieved by the database searches (Figure 1). Agreement between reviewers at title and abstract screening was 96.3% (κ = 0.67, substantial). Two further duplicates were found during screening, and 928 records were excluded. Seventy-eight reports were sought, including one that both reviewers had excluded at title and abstract screening and that was later reinstated as a report of an included study (Figure 1); 13 could not be retrieved. Of the 65 reports assessed in full, 58 were excluded: 50 for the wrong exposure, three for the wrong publication type, two for the wrong population, two because the full text was not in English, and one that duplicated another report (Table 10 in the Appendices). For the 64 reports first assessed in full, agreement between reviewers on eligibility was 98.4% (κ = 0.93), and AE adjudicated two decisions. The remaining seven reports described five new studies [38-44], because three reports drew on the same 29 staff interviews at one hospital [40-42]. One of these, a secondary analysis of the interviews by Germack et al., had been excluded at title and abstract screening and was reinstated when we checked for linked reports of included studies [41]. In total, 24 studies reported in 28 articles were included.

Study Characteristics

The included studies were published between 2005 and 2026 (Table 1). Ten were from the United States, four from Australia, two each from Canada and England, and one each from China, India, Japan, Saudi Arabia, Singapore, and Sweden. Twelve were qualitative studies using interviews, focus groups, observation, or ethnography [24,25,30-43], including one qualitative analysis of open-text survey responses [25]. Six were cross-sectional surveys [22,26-29,44], two were analyses of routinely collected records [21,23], one was a prospective observational cohort [17], one was a mixed-methods audit [18], and two were before-and-after quality improvement studies [19,20]. Sixteen studies collected data from clinicians, seven from patient records or hospital data, and one from patients and their receiving residents. Internal medicine was the most common inpatient specialty, and surgical specialties were the focus of only two studies [23,29].

**Table 1:** Characteristics of the included studies ED: emergency department. *Multiple specialties were general medicine, general surgery, short stay, cardiology, and orthopaedics [18]; medical and surgical specialties [21]; medicine, family medicine, cardiology, and critical care [26]; internal medicine and general surgery [32,33]; medicine and surgery [34]; internal medicine and surgical services [39]; medicine, surgery, orthopaedics, and neurosurgery [43]; and surgery, infectious diseases, internal medicine, orthopaedics, and others [44]. †Two reports of one study [32,33]. ‡Two reports of one study: the journal article [35] and the full project report [37]. §Three reports of one study [40-42]; only data on patients referred from the ED were extracted. The last five studies were identified by citation searching

Study	Country	Design	Setting	Participants or data	Additional data	Inpatient specialty	Type of dispute studied
Thaware et al., 2026 [17]	India	Prospective observational cohort	One tertiary teaching hospital	510 patients with ED stay of 24 hours or more	None	All specialties	Dispute or denial over lead specialty
Zhang et al., 2022 [18]	Australia	Mixed-methods audit	One tertiary teaching hospital	109 rejected admission requests	None	Multiple*	Rejected admission request
Shen and Lee, 2019 [19]	Singapore	Before-and-after quality improvement	One tertiary hospital	About 65,000 baseline admissions	None	All wards	Ward rejection of allocated beds
Lowthian et al., 2015 [20]	Australia	Before-and-after quality improvement	One tertiary hospital	215,125 ED presentations	None	Hospital-wide	Authority to admit within flow redesign
Iwata et al., 2013 [21]	Japan	Retrospective records review	Two tertiary EDs	1,215 consulted patients	None	Multiple*	Disposition disagreement
Wang et al., 2018 [22]	China	Cross-sectional hospital survey	18 teaching hospitals	3,169,958 ED visits over two years	None	Multiple	Refusal of complex patients
Cheng et al., 2021 [23]	United States	Retrospective records review	National federal violations database	98 vascular-related violations	None	Vascular surgery	Specialty refusal or unavailability
Caldicott et al., 2005 [24]	United States	Qualitative interviews	One teaching hospital	26 patients	Receiving residents’ interviews	Internal medicine	Perceived inappropriate transfer ("turfing")
Horwitz et al., 2009 [25]	United States	Qualitative analysis of open-text survey responses	One academic centre	139 physicians	36 incidents	Internal medicine	ED-to-inpatient transition failures
Smith et al., 2015 [26]	United States	Cross-sectional survey	One academic centre	126 physicians	None	Multiple*	Handoff conflict and responsibility
Lawrence et al., 2016 [27]	Australia	Cross-sectional survey	One tertiary hospital	72 trainees	None	Internal medicine	Admission expectations and refusal
Aldhibaib et al., 2025 [28]	Saudi Arabia	Cross-sectional survey	Four tertiary hospitals	104 emergency physicians	None	Multiple	Difficult referral
Cantu et al., 2012 [29]	United States	Cross-sectional survey	Regional EDs in two states	31 emergency physicians	None	Orthopaedics	Reluctance to attend
Kanjee et al., 2021 [30]	United States	Qualitative focus groups	One academic centre	35 physicians	None	Internal medicine	Disposition disagreement at handover
Schrepel et al., 2022 [31]	United States	Qualitative interviews	One academic institution	18 physicians	None	Internal medicine	Conflict during admission conversations
Chan et al., 2013 and 2014 [32,33]†	Canada	Qualitative interviews and focus groups	Four academic hospitals	61 physicians	None	Multiple*	Conflict during consultation
Lawrence et al., 2015 [34]	Australia	Qualitative interviews	One teaching hospital	25 clinicians	None	Multiple*	Power to accept or reject referrals
Sujan et al., 2014 and 2015 [35,37]‡	England	Qualitative observation and interviews	Three hospitals and two ambulance services	270 handovers	39 interviews	Acute medicine	Competing priorities at handover
Ghaseminejad et al., 2025 [36]	Canada	Qualitative focus groups	Two academic hospitals	41 physicians	None	General internal medicine	Interdepartmental conflict over admission
Apker et al., 2007 [38]	United States	Qualitative interviews	One tertiary teaching hospital	12 physicians	Brief observation before interviews	Hospital medicine	Disagreement over readiness for admission
Hilligoss, 2014 [39]	United States	Qualitative ethnography and discourse analysis	One academic tertiary hospital	86 participants; 48 interviews	349 hours of observation; 48 recorded handoffs	Multiple*	Negotiation, pushback and blocking of admissions
Rosenberg et al., 2018, Germack et al., 2020 and Fekieta et al., 2021 [40-42]§	United States	Qualitative ethnography and interviews	One academic tertiary hospital	29 staff (28 interviews)	31 hours of observation	General medicine and specialties	Specialty refusal and redirection to general medicine
Boiko et al., 2021 [43]	England	Qualitative interviews	One tertiary trust	19 staff	None	Multiple*	Referral conflict with admitting specialties
Martin Schilling, 2014 [44]	Sweden	Cross-sectional survey	One university hospital ED	18 emergency physicians	None	Multiple*	Turfing

Fifteen studies addressed admission disputes within a broader topic, such as crowding, handover, consultation, patient flow, or burnout; the data that made each study eligible are listed in the Appendices (Table 9). Only one study reported outcomes for patients known to have had a disputed or rejected referral [18]. Two further studies identified disputes or refusals within a wider group of patients but reported outcomes for the whole group rather than separately for the patients affected [17,23]. The remaining quantitative studies measured clinician perceptions, disposition agreement or system-level waits, and the qualitative studies described clinicians’ and patients’ accounts. Most of the evidence in this review therefore comes from clinicians’ reports.

Methodological Quality

MMAT ratings are summarised in Table 2. For the five studies identified by citation searching, the two reviewers’ MMAT judgements agreed on all 35 criteria. All 12 qualitative studies met all five qualitative criteria, although most drew on a single institution and a convenience or volunteer sample. The quantitative descriptive studies were limited mainly by uncertain representativeness, nonresponse and reliance on self-report; in one survey the proportion of patients affected by turfing was estimated by the respondents rather than counted [44], and in another the statistical analysis was judged inadequate for the research question [28]; none compared patients with and without a disputed referral. The two quality improvement studies used historical comparisons without concurrent controls or adjustment for confounding. The mixed-methods audit relied on clinician nomination of cases, which is likely to have missed some rejected referrals. Taken together, the audit and the two record-based studies [17,18,23] and the two quality improvement studies [19,20] provide the only measured data, but these are descriptive or uncontrolled; the qualitative studies provide the most detailed evidence on how disputes arise; and the surveys estimate how often clinicians perceive problems, with estimates that are vulnerable to nonresponse and recall bias.

**Table 2:** Methodological quality of the included studies using the MMAT 2018 MMAT: Mixed Methods Appraisal Tool S1 and S2 are the MMAT screening questions. Criteria 1 to 5 are the five criteria for each study’s design category [13]. No overall score is calculated, in line with MMAT guidance. The two reports of the Chan et al. study [32,33], the two reports of the Sujan et al. study [35,37], and the three reports of the Rosenberg et al. study [40-42] were each appraised together. For the mixed-methods study [18], the components were also appraised against their own criteria: qualitative component (criteria 1.1 to 1.5) Yes, Yes, Yes, Can’t tell, Yes; quantitative descriptive component (criteria 4.1 to 4.5) Yes, No, Can’t tell, No, Yes

Study	Category	S1	S2	Criterion 1	Criterion 2	Criterion 3	Criterion 4	Criterion 5
Caldicott et al., 2005 [24]	Qualitative	Yes	Yes	Yes	Yes	Yes	Yes	Yes
Horwitz et al., 2009 [25]	Qualitative	Yes	Yes	Yes	Yes	Yes	Yes	Yes
Kanjee et al., 2021 [30]	Qualitative	Yes	Yes	Yes	Yes	Yes	Yes	Yes
Schrepel et al., 2022 [31]	Qualitative	Yes	Yes	Yes	Yes	Yes	Yes	Yes
Chan et al., 2013 and 2014 [32,33]	Qualitative	Yes	Yes	Yes	Yes	Yes	Yes	Yes
Lawrence et al., 2015 [34]	Qualitative	Yes	Yes	Yes	Yes	Yes	Yes	Yes
Sujan et al., 2014 and 2015 [35,37]	Qualitative	Yes	Yes	Yes	Yes	Yes	Yes	Yes
Ghaseminejad et al., 2025 [36]	Qualitative	Yes	Yes	Yes	Yes	Yes	Yes	Yes
Apker et al., 2007 [38]	Qualitative	Yes	Yes	Yes	Yes	Yes	Yes	Yes
Hilligoss, 2014 [39]	Qualitative	Yes	Yes	Yes	Yes	Yes	Yes	Yes
Rosenberg et al., 2018, Germack et al., 2020 and Fekieta et al., 2021 [40-42]	Qualitative	Yes	Yes	Yes	Yes	Yes	Yes	Yes
Boiko et al., 2021 [43]	Qualitative	Yes	Yes	Yes	Yes	Yes	Yes	Yes
Shen and Lee, 2019 [19]	Non-randomised	Yes	Yes	Can't tell	Yes	Can't tell	No	Yes
Lowthian et al., 2015 [20]	Non-randomised	Yes	Yes	Yes	Yes	Can't tell	No	Can't tell
Thaware et al., 2026 [17]	Quantitative descriptive	Yes	Yes	Yes	Yes	Can't tell	Yes	Can't tell
Iwata et al., 2013 [21]	Quantitative descriptive	Yes	Yes	Yes	Yes	Can't tell	Yes	Yes
Wang et al., 2018 [22]	Quantitative descriptive	Yes	Yes	Yes	Yes	Can't tell	Can't tell	Yes
Cheng et al., 2021 [23]	Quantitative descriptive	Yes	Yes	Yes	Can't tell	Can't tell	Can't tell	Yes
Smith et al., 2015 [26]	Quantitative descriptive	Yes	Yes	Yes	Can't tell	Yes	Can't tell	Yes
Lawrence et al., 2016 [27]	Quantitative descriptive	Yes	Yes	Yes	Can't tell	Yes	Can't tell	Can't tell
Aldhibaib et al., 2025 [28]	Quantitative descriptive	Yes	Yes	Yes	Can't tell	Yes	Can't tell	No
Cantu et al., 2012 [29]	Quantitative descriptive	Yes	Yes	Yes	Can't tell	Can't tell	Can't tell	Can't tell
Martin Schilling, 2014 [44]	Quantitative descriptive	Yes	Yes	Yes	Can't tell	Can't tell	Can't tell	Yes
Zhang et al., 2022 [18]	Mixed methods	Yes	Yes	Yes	Yes	Can't tell	Yes	No

Measured Patient Outcomes

Measured outcomes are summarised in Table 3, grouped by delay, disposition, hospital stay, and mortality; no study reported adverse events in patients with a disputed referral. In a prospective cohort at a tertiary centre in India, 510 of 5,922 ED admissions (8.6%) remained in the ED for 24 hours or more without transfer [17]. Disputes or denials between specialties over primary responsibility were recorded in 166 of these 510 patients (32.5%), almost as often as waits for an intensive care or high-dependency bed (169, 33.1%). Among the 438 patients eventually transferred, median ED length of stay was 51 hours and in-hospital mortality was 16.2%; outcomes were not reported separately for patients with a dispute.

**Table 3:** Measured outcomes in studies reporting patient or system data ED: emergency department; CI: confidence interval; NR: not reported and not calculable from reported data; NA: not applicable CIs for proportions were calculated by the reviewers from reported counts with the Wilson score method. †As reported by the study. Only Zhang et al. [18] reported outcomes for patients known to have had a rejected referral. Thaware et al. [17] and Cheng et al. [23] reported outcomes for the whole group, not separately for patients with a dispute or refusal. Shen and Lee [19] and Lowthian et al. [20] were before-and-after studies without concurrent controls. No included study reported time from referral to acceptance for patients with and without a disputed referral, or adverse events verified against patient records. *Excluding patients admitted to the ED short-stay unit

Study	Outcome domain	Measure	Group or period	Value	95% CI
Thaware et al., 2026 [17]	Exposure	Dispute over lead specialty	Prolonged boarders (n = 510)	166 (32.5%)	28.6% to 36.7%
Thaware et al., 2026 [17]	Delay	Median ED length of stay	Transferred (n = 438)	51 hours	NR
Thaware et al., 2026 [17]	Mortality	In-hospital mortality	Transferred (n = 438)	71 (16.2%)	13.1% to 20.0%
Thaware et al., 2026 [17]	Mortality	In-hospital mortality	Not transferred (n = 72)	13 (18.1%)	10.9% to 28.5%
Zhang et al., 2022 [18]	Exposure	Rejected admission requests	Audited patients	109	NA
Zhang et al., 2022 [18]	Delay	Median ED length of stay	Rejected requests (n = 109)	9 hours	NR
Zhang et al., 2022 [18]	Delay	Range of ED length of stay	Rejected requests (n = 109)	0 to 37 hours	NA
Zhang et al., 2022 [18]	Delay	Correlation of ED length of stay with number of teams (Spearman ρ)	Rejected requests (n = 109)	0.409 (p < 0.001)	NR
Zhang et al., 2022 [18]	Disposition	Eventually admitted	Rejected requests (n = 109)	97 (89.0%)	81.7% to 93.6%
Zhang et al., 2022 [18]	Disposition	Admitted under first team contacted	Rejected requests (n = 109)	66 (60.6%)	51.2% to 69.2%
Zhang et al., 2022 [18]	Hospital stay	Hospital stay longer than seven days	Admitted (n = 97)	24 (24.7%)	17.2% to 34.2%
Cheng et al., 2021 [23]	Exposure	Specialty refusal or unavailability	Violations (n = 98)	30 (30.6%)	22.4% to 40.3%
Cheng et al., 2021 [23]	Disposition	Transferred to another facility	Violations (n = 98)	41 (41.8%)	32.6% to 51.7%
Cheng et al., 2021 [23]	Mortality	Overall mortality	Violations (n = 98)	19 (19.4%)	12.8% to 28.3%
Cheng et al., 2021 [23]	Mortality	Deaths during index ED visit	Violations (n = 98)	6 (6.1%)	2.8% to 12.7%
Iwata et al., 2013 [21]	Exposure	Disposition disagreement	Consulted patients (n = 1,215)	62 (5.1%)	4.0% to 6.5%
Iwata et al., 2013 [21]	Disposition	Agreement on disposition (κ)	Consulted patients (n = 1,215)	0.76	0.70 to 0.82†
Iwata et al., 2013 [21]	Disposition	Disagreement without initial ED diagnosis	Discordant cases (n = 62)	39 (62.9%)	50.5% to 73.8%
Wang et al., 2018 [22]	Delay	Mean time boarded before admission	2013	37.1 hours	18.0 to 56.1 hours†
Wang et al., 2018 [22]	Delay	Mean time boarded before admission	2014	36.2 hours	18.2 to 54.2 hours†
Wang et al., 2018 [22]	Delay	Boarded patients as a share of ED visits	2013	2.7%	NR
Wang et al., 2018 [22]	Delay	Boarded patients as a share of ED visits	2014	2.6%	NR
Shen and Lee, 2019 [19]	Exposure	Bed allocations rejected by wards	Before	9%	NR
Shen and Lee, 2019 [19]	Exposure	Bed allocations rejected by wards	After	5%	NR
Shen and Lee, 2019 [19]	Delay	ED bed request to acknowledgement	Before	2 h 10 min	NR
Shen and Lee, 2019 [19]	Delay	ED bed request to acknowledgement	After	47 min	NR
Shen and Lee, 2019 [19]	Delay	Disposition to ward arrival	Before	4 h 18 min	NR
Shen and Lee, 2019 [19]	Delay	Disposition to ward arrival	After	2 h 43 min	NR
Shen and Lee, 2019 [19]	Delay	Additional wait per rejection	Whole period	About 45 min	NR
Lowthian et al., 2015 [20]	Delay	Median ED length of stay, admitted patients	Before (2010)	437 min	NR
Lowthian et al., 2015 [20]	Delay	Median ED length of stay, admitted patients	After (2013)	346 min	NR
Lowthian et al., 2015 [20]	Delay	Four-hour target met	Before (2010)	60%	NR
Lowthian et al., 2015 [20]	Delay	Four-hour target met	After (2013)	74%	NR
Lowthian et al., 2015 [20]	Mortality	In-hospital mortality, patients admitted through the ED*	Before	3.5%	NR
Lowthian et al., 2015 [20]	Mortality	In-hospital mortality, patients admitted through the ED*	After	2.2%	NR

An Australian audit of 109 ED patients whose admission request was initially rejected found that 97 (89.0%) were eventually admitted and 66 (60.6%) were admitted under the team first contacted [18]. Median ED length of stay was nine hours (range: 0-37) and increased with the number of inpatient teams involved (Spearman ρ = 0.409; p < 0.001). The commonest reason recorded for a rejection was the inpatient team directing the admission to another team, followed by requests to review the patient in person, disagreement about clinical decisions, and requests for further investigations.

In a United States analysis of 98 vascular-related violations of the Emergency Medical Treatment and Labor Act, specialty refusal or unavailability was the most frequent reason (30 cases, 30.6%), and 41 patients (41.8%) were transferred to another facility [23]. Overall mortality was 19.4% (19 of 98), including six deaths during the index ED visit, but outcomes were not separated for refusal cases. In two Japanese EDs, emergency physicians and consultants disagreed about disposition in 62 of 1,215 consulted patients (5.1%; κ = 0.76), and 39 of these 62 patients (63%) had no initial ED diagnosis [21]. In 18 Beijing teaching hospitals, inpatient doctors’ refusal of complex or multisystem patients was described as one cause of prolonged observation stays, and boarded patients waited a mean of 36 to 37 hours for admission [22].

Two studies evaluated hospital-wide changes intended to reduce refusals or clarify responsibility. In Singapore, a programme that redefined ward categories and introduced a policy of no arbitrary rejection of allocated beds was followed by a fall in the proportion of bed allocations rejected by wards from 9% to 5% [19]. For ED patients, mean time from bed request to acknowledgement fell from two hours 10 minutes to 47 minutes, and mean time from disposition to ward arrival fell from four hours 18 minutes to two hours 43 minutes; each rejection added about 45 minutes to the wait. In Melbourne, a hospital-wide redesign that included early assignment of management responsibility and emergency physician authority to admit was followed by a fall in median ED length of stay for admitted patients from 437 to 346 minutes and in in-hospital mortality among patients admitted through the ED from 3.5% to 2.2% [20]. Neither study isolated the effect of the dispute-related components, and neither had a concurrent control.

The statistics in Table 3 describe the groups studied and do not estimate the effect of a dispute. The correlation between the number of teams involved and ED length of stay [18] was unadjusted for illness severity, came from clinician-nominated cases, and could reflect longer stays leading to more teams being contacted as well as the reverse. The κ statistic [21] measures agreement on disposition across all consulted patients, not outcomes after disagreement. Several CIs are wide because the groups were small.

Patient-Reported Experience

Only one study asked patients about their experience [24]. In a United States teaching hospital, receiving medical residents’ accounts were used to classify 26 transferred or admitted patients as "turf" admissions (eight), appropriate admissions (16) or equivocal (two); the classification reflected the receiving residents’ perception that a transfer was inappropriate, not the patients’ own view. Patients whose admissions the residents regarded as turfed described predominantly unfavourable experiences, including anger and frustration, whereas the remaining patients gave mixed accounts. Most of the patients classed as turfed had come through the ED. The design could not show whether the transfer caused the poorer experience or whether patients perceived as unwanted were also more difficult to care for.

Clinician-Reported Consequences for Patients

Clinicians in all 16 studies that collected data from clinicians described consequences for patients; three analytical themes emerged. These consequences were reported by clinicians and were not verified against patient records. The analytical themes for consequences, contributing factors and mitigating strategies, the studies contributing to each, and our confidence in each finding are summarised in Table 4.

**Table 4:** Summary of qualitative findings and confidence in each finding (GRADE-CERQual) ED: emergency department; MMAT: Mixed Methods Appraisal Tool Confidence was assessed with GRADE-CERQual [16], considering methodological limitations, coherence, adequacy of data, and relevance. The reports of the Chan et al. study [32,33] and of the Rosenberg et al. study [40-42] are each counted as one study

Theme	Summary of review finding	Contributing studies	Confidence	Explanation of confidence judgement
Patient experience	Patients whom receiving residents regarded as inappropriately transferred described poorer experiences of care	[24]	Very low	One small study with residents’ rather than patients’ classification; data more than 20 years old
Delay and repeated referral	Patients waited while teams disagreed about admission, placement or tests before acceptance, and were redirected between specialties	[18,28-31,38,40,42-44]	Moderate	Consistent across nine studies in five countries, including one audit with measured ED length of stay; mostly self-reported, single-centre data
Risk to patient safety	Clinicians recalled diagnostic, treatment and disposition errors and deterioration after contested or unclear transfer, and regarded turfing as dangerous	[25-27,38,44]	Low	Recalled or perceived, unverified events from five single-centre studies; several concern ED-to-inpatient transfer broadly, not only disputes
Loss of focus on the patient	Negotiation shifted from the patient’s needs to which team should take responsibility, and could end with the patient admitted under the team that gave way rather than the most appropriate team	[26,34,36,39-43]	Low	Six studies, but few specific examples of patients placed under a less appropriate team and none verified against records; in one study the main focus was burnout
Clinical uncertainty	Patients without an ED diagnosis, or with complex or multisystem problems, were more often the subject of disagreement or refusal, and inpatient teams sometimes required a completed workup before accepting	[18,21,22,38,40,43]	Moderate	Coherent findings supported by record-based data in two studies and clinicians’ accounts in four; few centres
System pressure	Bed shortages, crowding, time targets and workload contributed to disputes	[17,22,35,36,40,43]	Moderate	Consistent across six studies in five countries and different designs; largely descriptive
Unclear or unequal admission rules	There was no explicit rule on when responsibility transferred from the ED to the admitting team, and some services could decline admissions while general medicine could not	[25,26,39-42]	Moderate	Coherent findings from four studies, including two ethnographies with rich data; all were single-centre studies from the United States, and two addressed handoff broadly
Referral timing	Referrals at night, at weekends and near shift handover were more often contested	[28,29]	Low	Two surveys with self-reported data and possible nonresponse bias
Professional identity and trust	Specialty allegiance, hierarchy, stereotypes, previous negative encounters and perceived challenges to expertise shaped admission conversations	[30-33,38,39,43]	Moderate	Rich, coherent data from six qualitative studies meeting all MMAT criteria; academic centres in three countries
Proposed mitigating strategies	Clinicians proposed explicit admission rules, shared pathways, structured referral communication, joint training, collaborative handoffs and senior mediation; an existing escalation route was often bypassed	[20,25,26,30-33,35,39,41-43]	Low	Proposals were not evaluated; the only evaluations were two uncontrolled before-and-after studies of multicomponent changes

Delay and repeated referral: Clinicians described patients waiting while teams disagreed about the need for admission, ward, or intensive care placement, or investigations required before transfer [30,31]. In Saudi Arabia, 94 of 104 emergency physicians (90.4%) reported difficulty with referrals; 86 (82.7%) had been redirected to another specialty before the patient was seen; 79 (76.0%) had a referral refused at the time of shift handover; and 76 (73.1%) had been asked for additional tests before acceptance [28]. In northern New England, 55% of emergency physicians reported that the on-call orthopaedic surgeon was reluctant to attend, only 29% said the surgeon always came when asked, and 52% believed patient care could often have been improved by orthopaedic assessment [29]. In a United States teaching hospital, one hospitalist described never accepting a patient until all test results were back and the workup was complete, which emergency physicians said lengthened patients’ waits; in one recalled case, a patient with sepsis waited for hours in the ED while the intensive care team refused admission, until a hospitalist’s assessment led to acceptance [38]. Emergency doctors in London described specialists asking them to call back with blood results or a computed tomography scan before accepting a patient, and having to defend their referrals [43]. At a United States academic centre, a specialty that declined an ED patient could direct the ED to book the patient to general medicine instead [40], and such refusals were usually not escalated as hospital protocol required; the patient was instead admitted to general medicine as "the path of least resistance" [42]. In a Swedish university hospital ED, all 18 responding emergency physicians reported that turfing occurred, estimating that it affected a mean of 17.3% of their patients (range 2% to 50%), and 17 (94.4%) believed that turfing and "optimising" (presenting patients’ symptoms so that investigations would be approved) lengthened ED waits [44]. These percentages are respondents’ estimates rather than counts of patients.

Risk to patient safety: In a qualitative analysis of open-text survey responses at one United States academic centre, 40 of 139 responding physicians (29%) reported that a patient had experienced an adverse event or near miss after transfer from the ED to internal medicine [25]. These respondents described 36 errors, of which 13 involved diagnosis, 14 treatment, and 13 dispositions; some errors fell into more than one category, so the category counts sum to more than 36. Six patients in these accounts required escalation from the ward to intensive care within 24 hours. In a separate cross-sectional survey at another United States academic centre, 36 of 126 physicians (29%) reported at least one handoff-related adverse event or near miss in the previous three months [26]. Internal medicine trainees in Australia were much less comfortable than ED trainees with admission without inpatient review (13 of 56, 23.2%, compared with 14 of 16, 87.5%) and viewed direct admission as a safety threat [27]. In the Swedish survey, 12 of 18 emergency physicians (67%) regarded turfing and optimising as unsafe for the patient, and none regarded them as safe [44]. These events and judgements were self-reported and were not verified against records.

Loss of focus on the patient: Clinicians described negotiations in which the conversation became about which team should take responsibility rather than what the patient needed [34,36]. Physicians felt defensive during these exchanges, with 94% of emergency respondents in one survey reporting feeling defensive at least sometimes [26]. In Canada, internal medicine physicians described being pulled into a "turf war" over a patient who "bounces" between teams and whom no team wants to admit, which they linked to burnout and reduced capacity to build rapport with patients [36]. In a two-year ethnography at a United States tertiary hospital, doctors used the language of selling, pushing back and "blocking" to describe admission handoffs; one emergency attending observed that when such a dispute was resolved, "it doesn’t necessarily mean the patient goes to the best service, but maybe just the weakest personality caves" [39]. Hospitalists at another United States centre said that patients did not always go to the right service because specialties could redirect them to general medicine [40], and patients who needed care the receiving unit could not provide, such as telemetry, were re-booked to a different unit [41]. ED staff in London described compromises over the placement of complex patients that led to later moves between wards, which were difficult for patients and wards [43].

Contributing Factors

Five groups of contributing factors were reported. The first was clinical uncertainty: patients without an ED diagnosis accounted for most disposition disagreements [21], complex, multimorbid, or deteriorating patients were more often declined [18,22,40], and some inpatient teams required a completed workup before accepting a patient [38,43]. The second was system pressure, including bed shortages, crowding, time targets, and workload [17,22,35,36,40,43]. The third was unclear or unequal admission rules. There was no explicit institutional rule for when responsibility transferred from the ED to the admitting team [25,26], and in two United States hospitals specialties could decline admissions while general medicine could not, so that patients declined by a specialty defaulted to general medicine [39,40,42]. In one of these hospitals, the ED could book patients to general medicine without the receiving team’s acceptance, whereas specialties such as urology required approval, and responsibility for the patient passed from the ED physician at the four-hour mark [41]. The fourth was timing, with referrals at night, at weekends and near shift handover more often contested [28,29]. The fifth was professional identity and trust: physicians described in-group allegiance to their specialty, stereotypes of other specialties, previous negative encounters and perceived challenges to their expertise [30-33], differing views between emergency physicians and hospitalists of what an admission required [38], and hierarchy, with junior specialists reluctant to accept admissions that their consultants might consider inappropriate [43]. Admission handoffs were described in the language of selling and competition [39].

Mitigating Strategies

No study evaluated an intervention targeting inter-specialty disputes alone. Clinicians proposed explicit admission rules and clear allocation of responsibility [20,25,26], shared pathways for common presentations agreed between departments [30], structured referral communication, such as the PIQUED model of preparation, identification, questions, urgency, educational modifications and discussion [33], and opportunities for emergency and inpatient teams to train and work together [31,32,35]. Some doctors described handoffs framed as teamwork, in which differing perspectives were discussed and a disposition agreed, as an alternative to competitive negotiation [39], and ED staff described mediating between specialties over complex patients [43]. One hospital had a protocol for escalating specialty refusals to the leadership on duty, but refusals were usually not escalated [42], and conflict over transfers was described as simmering at staff level rather than reaching administrators, although one emergency physician said a joint ED and medicine huddle helped open communication [41]. The two quality improvement studies measured waits before and after hospital-wide changes that included a policy against arbitrary bed rejection and emergency physician authority to admit [19,20]; their results are given in Table 3.

Discussion

Principal Findings

The existing studies cannot estimate the effect of disputed admission referrals on patient outcomes, but they consistently link disputes to delay and repeated referral, mostly through clinicians’ accounts. Measured data were limited to a single audit of rejected referrals and to studies that counted disputes without reporting outcomes for the patients affected. Clinicians described disputes as arising from the interaction of diagnostic uncertainty, system pressure, unclear or unequal rules on responsibility and professional identity, rather than from individual behaviour alone. Our confidence in these findings was at most moderate.

Comparison With Previous Work

Previous reviews have shown that ED crowding is associated with harm [1] and that prolonged boarding has been linked to higher in-hospital mortality, although inconsistently across studies [6]. These reviews treat the wait for an inpatient bed as a single interval and attribute it mainly to bed capacity. A systematic review of ED consultation described how often consultations occur and how often consulted patients are admitted, but not what happens when the consulting team declines [7]. A review of handoff failures during transfers within hospitals, including from the ED to the ward, focused on communication and information loss [8], and an umbrella review of interventions to improve ED patient flow found weak evidence for most interventions [9].

This review adds a distinct step to that literature: the period between the ED’s decision to refer and an inpatient team’s acceptance, during which responsibility may be contested. Disputes over responsibility, as distinct from bed availability or information transfer, have not previously been synthesised. Ethnographic studies describe admission handoffs as negotiations in which services hold unequal authority to refuse [39,40], which locates part of the problem in hospital rules rather than only in communication between individuals. The included studies suggest that this step is common among patients with very long ED stays [17] and that it lengthens ED stays when several teams are approached [18], yet neither the crowding literature nor the evaluations of flow interventions measure it separately. In the only audit of rejected referrals, most patients were eventually admitted, usually under the team first contacted, which suggests that disputes more often delay than prevent admission [18]. This is consistent with descriptions of turfing as a disagreement about placement during which no team takes ownership [4], and with the finding that patients may notice when they are regarded as unwanted [24]. Professional disagreement can improve care when it is resolved openly and quickly [5]; the included studies suggest that the risk lies in disagreement that is unstructured, unresolved, and conducted while no team holds clear responsibility.

Implications for Practice

For EDs and hospital managers, the main implication is that disputed referrals are largely invisible in routine data. Recording the number of teams contacted and the time from first referral to acceptance, as the Australian audit did [18], would show how often disputes occur locally and how long they last, at little cost. Where disputes are frequent, the measures clinicians proposed address the causes they described: explicit rules on which team accepts patients who fall between specialties and when responsibility transfers, a senior escalation route for contested referrals whose use is monitored, since one hospital’s route was usually bypassed [42], and a clear statement at each handoff of which team is responsible until the referral is accepted. Because disputes concentrated in patients without a clear diagnosis or with complex needs [18,21,22], these are the patients for whom an agreed default admitting team is most relevant. Hospital-wide changes that included such rules were followed by shorter waits [19,20], but they were uncontrolled and multicomponent, so any local change should be introduced and evaluated as a quality improvement project rather than assumed to work.

Implications for Research

Future research should record dispute exposure prospectively, for example, as the number of teams contacted and the time from first referral to acceptance, and link it to patient outcomes such as ED length of stay, adverse events, and mortality, with adjustment for illness severity. Patient experience of disputed referrals deserves direct study, since it was examined by only one small study published two decades ago. Findings rated low or very low confidence, particularly the risk to patient safety and the role of referral timing, need confirmation with verified rather than recalled events. Agreement on a standard definition of a disputed referral would allow studies to be compared and combined. Because studies in this field have appeared at a rate of about one a year and none has yet linked dispute exposure to patient outcomes, an update of this review will be most useful once such prospective studies are published; a living review, reported with the PRISMA extension for living systematic reviews [45], could be considered if they begin to appear regularly.

Strengths and Limitations

This review followed a registered protocol, searched four databases with subject headings and free text, tested the strategy against known studies, supplemented the searches with backward and forward citation searching, used independent dual screening of titles and abstracts and, for studies from the database searches, independent dual extraction and appraisal, and assessed confidence in qualitative findings with GRADE-CERQual. It also has limitations. Searches were restricted to databases available through National Health Service institutional access; Scopus was used only for forward citation searching, and Web of Science was not searched. Because "turfing" and related terms are colloquial and inconsistently indexed, some studies may still have been missed: citation searching, added after peer review, identified five further studies that the database searches had not found, and it was limited to a single round. English-language studies only were included; two reports were excluded because the full text was not in English, and 14 reports could not be retrieved. For reports identified by citation searching, full-text assessment, data extraction, and appraisal were checked by two reviewers. Agreement between reviewers at title and abstract screening of database records was moderate, reflecting a broad and inconsistently described exposure. The included studies were small, mostly single-centre and largely descriptive, used different definitions of a dispute, and drew mainly on internal medicine in high-income countries. Most of the evidence came from clinicians’ reports rather than patient-level data; many studies relied on clinicians’ recall, and several included broader handover, consultation, or flow problems in which disputes were one element. The findings should therefore be read as describing associations and mechanisms rather than estimating the effect of disputes on patient outcomes.

## Conclusions

Disputes over which inpatient team accepts an ED admission are commonly described by clinicians, rarely measured, and a potentially modifiable source of delay. Recording the number of teams contacted and the time from first referral to acceptance would allow hospitals to measure the problem and to test whether clearer admission rules shorten it. The current evidence, which comes mostly from clinicians’ reports, cannot yet estimate the effect of disputes on patient outcomes, and prospective studies that link dispute exposure to patient outcomes are needed.

## Appendices

Appendices

Search strategies, all run on 12 October 2026.

**Table 5:** Ovid MEDLINE(R) ALL, 1946 to 9 October 2026. Limit: English language. Records retrieved: 614

Line	Search	Results
1	(turfing or turfed).ti,ab.	30
2	(ownership adj3 (patient* or care)).ti,ab.	763
3	(most responsible physician or most responsible clinician or diffusion of responsibility).ti,ab.	138
4	(responsib adj3 (disput or contest or conflict or disagree or refus or defer or unclear or ambigu or diffus or shift)).ti,ab.	2,973
5	((interspecialt or inter-specialt or interdepartment or inter-department or between specialt or between team or between service) adj5 (conflict or disput or disagree or tension or friction or negotiat*)).ti,ab.	100
6	((consult or referral or admission or admit) adj3 (refus or declin or reject or disput or resist or contest or negotiat*)).ti,ab.	2,737
7	((conflict or disagree or friction or disput or tension) adj5 (handover or hand-over or handoff or hand-off or admission or admit* or disposition)).ti,ab.	262
8	Interprofessional Relations/ and (responsib or ownership or refus or disput*).ti,ab.	2,560
9	"Referral and Consultation"/ and (refus or disput or disagree or conflict or declin*).ti,ab.	2,890
10	or/1-9	11,982
11	exp Emergency Service, Hospital/ or Emergency Medicine/	128,932
12	(emergency department or emergency room or emergency medicine or "accident and emergency" or emergency physician or emergency unit or emergency admission*).ti,ab.	215,980
13	11 or 12	260,183
14	10 and 13	638
15	limit 14 to english language	614

**Table 6:** Embase (Ovid), 1974 to 8 October 2026. Limits: English language; conference abstracts removed. Records retrieved: 918 A further six preprints and 99 trial registry records were removed from the Embase results before screening

Line	Search	Results
1	(turfing or turfed).ti,ab.	34
2	(ownership adj3 (patient* or care)).ti,ab.	1,129
3	(most responsible physician or most responsible clinician or diffusion of responsibility).ti,ab.	227
4	(responsib adj3 (disput or contest or conflict or disagree or refus or defer or unclear or ambigu or diffus or shift)).ti,ab.	3,609
5	((interspecialt or inter-specialt or interdepartment or inter-department or between specialt or between team or between service) adj5 (conflict or disput or disagree or tension or friction or negotiat*)).ti,ab.	172
6	((consult or referral or admission or admit) adj3 (refus or declin or reject or disput or resist or contest or negotiat*)).ti,ab.	4,964
7	((conflict or disagree or friction or disput or tension) adj5 (handover or hand-over or handoff or hand-off or admission or admit* or disposition)).ti,ab.	391
8	interdisciplinary communication/ and (responsib or ownership or refus or disput*).ti,ab.	605
9	exp patient referral/ and (refus or disput or disagree or conflict or declin*).ti,ab.	9,887
10	or/1-9	20,059
11	exp emergency ward/ or emergency medicine/ or emergency health service/	443,438
12	(emergency department or emergency room or emergency medicine or "accident and emergency" or emergency physician or emergency unit or emergency admission*).ti,ab.	354,838
13	11 or 12	540,439
14	10 and 13	1,642
15	limit 14 to english language	1,609
16	15 not conference abstract.pt.	918

**Table 7:** CINAHL (EBSCOhost). Search mode: Boolean/Phrase; "Apply equivalent subjects" on. Limit: English language. Records retrieved: 445 CINAHL: Cumulative Index to Nursing and Allied Health Literature S15 adds the "turfing" terms (S1) without the emergency setting limit, because they are highly specific and a known relevant record lacked emergency terms in its CINAHL record

Line	Search	Results
S1	TI (turfing OR turfed) OR AB (turfing OR turfed)	10
S2	TI (ownership N3 (patient OR care)) OR AB (ownership N3 (patient OR care))	496
S3	TI ("most responsible physician" OR "most responsible physicians" OR "most responsible clinician" OR "most responsible clinicians" OR "diffusion of responsibility") OR AB ("most responsible physician" OR "most responsible physicians" OR "most responsible clinician" OR "most responsible clinicians" OR "diffusion of responsibility")	62
S4	TI (responsib N3 (disput OR contest OR conflict OR disagree OR refus OR defer OR unclear OR ambigu OR diffus OR shift)) OR AB (responsib N3 (disput OR contest OR conflict OR disagree OR refus OR defer OR unclear OR ambigu OR diffus OR shift))	1,343
S5	TI ((interspecialt OR inter-specialt OR interdepartment OR inter-department OR (between W0 (specialt OR team OR service))) N5 (conflict OR disput OR disagree OR tension OR friction OR negotiat)) OR AB ((interspecialt OR inter-specialt OR interdepartment OR inter-department OR (between W0 (specialt OR team OR service))) N5 (conflict OR disput OR disagree OR tension OR friction OR negotiat))	64
S6	TI ((consult OR referral OR admission OR admit) N3 (refus OR declin OR reject OR disput OR resist OR contest OR negotiat)) OR AB ((consult OR referral OR admission OR admit) N3 (refus OR declin OR reject OR disput OR resist OR contest OR negotiat))	1,279
S7	TI ((conflict OR disagree OR friction OR disput OR tension) N5 (handover OR hand-over OR handoff OR hand-off OR admission OR admit OR disposition)) OR AB ((conflict OR disagree OR friction OR disput OR tension) N5 (handover OR hand-over OR handoff OR hand-off OR admission OR admit OR disposition))	114
S8	(MH "Interprofessional Relations") AND (TI (responsib OR ownership OR refus OR disput) OR AB (responsib OR ownership OR refus OR disput))	1,672
S9	(MH "Referral and Consultation+") AND (TI (refus OR disput OR disagree OR conflict OR declin) OR AB (refus OR disput OR disagree OR conflict OR declin))	2,242
S10	S1 OR S2 OR S3 OR S4 OR S5 OR S6 OR S7 OR S8 OR S9	6,983
S11	(MH "Emergency Service+") OR (MH "Emergency Medicine")	92,812
S12	TI ((emergency W0 (department OR room OR physician OR unit OR admission)) OR "emergency medicine" OR "accident and emergency") OR AB ((emergency W0 (department OR room OR physician OR unit OR admission)) OR "emergency medicine" OR "accident and emergency")	101,502
S13	S11 OR S12	143,294
S14	S10 AND S13	439
S15	S1 OR S14	448
S16	S15 AND LA English	445

**Table 8:** Health Management Information Consortium (Ovid), 1979 to August 2026. No language limit available. Records retrieved: 18 CINAHL: Cumulative Index to Nursing and Allied Health Literature The Health Management Information Consortium database has its own thesaurus, so only free-text terms were used. Line 11 adds the "turfing" terms without the emergency setting limit, as in CINAHL

Line	Search	Results
1	(turfing or turfed).ti,ab.	1
2	(ownership adj3 (patient* or care)).ti,ab.	54
3	(most responsible physician or most responsible clinician or diffusion of responsibility).ti,ab.	4
4	(responsib adj3 (disput or contest or conflict or disagree or refus or defer or unclear or ambigu or diffus or shift)).ti,ab.	159
5	((interspecialt or inter-specialt or interdepartment or inter-department or between specialt or between team or between service) adj5 (conflict or disput or disagree or tension or friction or negotiat*)).ti,ab.	27
6	((consult or referral or admission or admit) adj3 (refus or declin or reject or disput or resist or contest or negotiat*)).ti,ab.	159
7	((conflict or disagree or friction or disput or tension) adj5 (handover or hand-over or handoff or hand-off or admission or admit* or disposition)).ti,ab.	7
8	or/1-7	405
9	(emergency department or emergency room or emergency medicine or "accident and emergency" or emergency physician or emergency unit or emergency admission*).ti,ab.	4,090
10	8 and 9	17
11	1 or 10	18

**Table 9:** Included studies with a broader focus and the data that made each eligible ED: emergency department Each study met the eligibility rule in the Methods because it reported data on an inpatient team disputing, declining, redirecting, or delaying acceptance of an ED admission referral; only those data were extracted

Study	Broader topic	Data used in this review
Shen and Lee, 2019 [19]	Admission waits and bed management	Wards rejecting beds allocated to admitted patients, including ED patients, which deferred acceptance at the point of admission
Lowthian et al., 2015 [20]	Hospital-wide patient flow redesign	Early assignment of management responsibility and emergency physician authority to admit, and waits before and after
Iwata et al., 2013 [21]	Agreement on disposition	Disagreement between emergency physicians and consultants about whether a patient should be admitted
Wang et al., 2018 [22]	ED overcrowding	Refusal by inpatient doctors to accept complex or multisystem patients, reported as a cause of prolonged ED observation
Cheng et al., 2021 [23]	Federal emergency-care law violations	The hospital’s own on-call specialist refusing or being unavailable to see ED patients; transfer to another facility was a consequence, not the exposure
Horwitz et al., 2009 [25]	ED-to-inpatient transition failures	Contested acceptance and unclear responsibility at the point of admission, and recalled harm after transfer
Smith et al., 2015 [26]	ED-to-inpatient handoffs	Disagreement with ED decisions, defensiveness and unclear responsibility during admission handoffs
Cantu et al., 2012 [29]	Orthopaedic on-call coverage	On-call surgeons’ reluctance to attend or decision to decline ED patients, and the reasons; transfer was a consequence, not the exposure
Chan et al., 2013 and 2014 [32,33]	ED consultation	Conflict, avoidance of consultation and negotiation over responsibility during requests for admission or further management
Sujan et al., 2014 and 2015 [35,37]	Handover across organisational boundaries	Negotiation and unclear responsibility at ED-to-acute medicine handover under capacity and target pressure
Ghaseminejad et al., 2025 [36]	Physician burnout	Interdepartmental disputes over which service would admit a patient as the most responsible physician
Apker et al., 2007 [38]	Emergency physician to hospitalist handoffs and patient safety	Hospitalists’ conditions for accepting admissions, disagreement over readiness for admission, and a recalled refusal by the intensive care team
Hilligoss, 2014 [39]	Language and interpretive frames in admission handoffs	Negotiation, pushback and blocking of admissions, and services’ differing authority to refuse admissions
Rosenberg et al., 2018, Germack et al., 2020 and Fekieta et al., 2021 [40-42]	Transfers within the hospital, including from the intensive care unit	Specialty refusal of ED patients and redirection to general medicine; only data on patients referred from the ED were extracted
Boiko et al., 2021 [43]	Interprofessional barriers in patient flow	Referral conflicts with admitting specialties, demands for investigations before acceptance, and mediation over complex patients

**Table 10:** Reports excluded after full-text review, with reasons (n = 77) ED: emergency department Database searches: 11 reports were excluded for the wrong exposure, six for the wrong publication type, two for the wrong population and one for the wrong outcome; one further report could not be retrieved. The project report by Sujan et al. [37] was initially excluded at full text and was later reclassified as a second report of an included study, so it is not listed here. Citation searching: 50 reports were excluded for the wrong exposure, three for the wrong publication type, two for the wrong population, and two because the full text was not in English; one further report was a duplicate citation of Kessler et al., 2012 (consultation). Thirteen reports from citation searching could not be retrieved: Benjamin et al., 2016; Berlyand et al., 2023; Claret et al., 2023; Gu and Itoh, 2019; Gu et al., 2017; Gurley et al., 2020; Kindermann et al., 2014; Kusnezov et al., 2020; Labaf et al., 2019; Nelson et al., 2017; Nie et al., 2024; Salter et al., 2023; and Stebbins and Hardman, 1993

Report	Identified by	Reason for exclusion	Explanation
Abo-Naser et al., 2026	Database searches	Wrong exposure	Effect of a new urgent care centre on upper limb trauma treated in the ED; no dispute over an admission referral
Charbonneau et al., 2018	Database searches	Wrong exposure	ED surge and end-of-shift effects on workup before referral to internal medicine; acceptance of the referral not studied
Dal Bo et al., 2025	Database searches	Wrong exposure	General patient-flow governance intervention; disputed or declined specialty admissions not studied
"ED/hospitalist plan improves throughput", 2009	Database searches	Wrong publication type	News item without a study design or outcome measurement
Goh et al., 2018	Database searches	Wrong exposure	Descriptive account of an acute medical unit; contested admission referrals not examined
Graf et al., 2025	Database searches	Wrong publication type	Simulation-based teaching case; not an empirical study of patients or clinicians
Haidet and Osorio, 2005	Database searches	Wrong publication type	Narrative essay (Health Affairs, Narrative Matters) recounting one patient’s disputed admission; not an empirical study
Hansen et al., 2026	Database searches	Wrong population	Child and youth mental health admissions; children only and mental-health-only referrals are excluded
Hou et al., 2026	Database searches	Wrong exposure	Emergency nurses’ experience of violence from patients and visitors
Jelinek et al., 2013	Database searches	Wrong exposure	Tensions in ED care of people with advanced cancer rather than disputes over admission responsibility
Leong, 2026	Database searches	Wrong publication type	Theoretical game-theory modelling without empirical data
Mgboji et al., 2021	Database searches	Wrong exposure	Incivility between ophthalmology and emergency medicine residents during consultation; no dispute over admission
Nugus et al., 2010	Database searches	Wrong outcome	Ethnography of ED boundary-work that described negotiation with inpatient teams over admission, but reported no patient outcomes, patient experience or clinician-reported consequences for patients
Salehi et al., 2014	Database searches	Wrong exposure	Clinical governance action research on ED length of stay without a specific disputed-admission exposure
Sandahl et al., 2024	Database searches	Wrong population	Pathways after self-harm; mental-health-only referrals are excluded
Schrepel et al., 2021	Database searches	Wrong publication type	Conference abstract; the full report of the same study is included [31]
Shefer et al., 2014	Database searches	Wrong exposure	Diagnostic overshadowing in patients with mental illness rather than contested admission responsibility
Stein, 2001	Database searches	Wrong publication type	Editorial
Wong et al., 2013	Database searches	Wrong exposure	Design and standardisation of a handover tool rather than admission refusal or responsibility disputes
Ygiyeva et al., 2023	Database searches	Wrong exposure	Paramedics consulting providers in the ED rather than ED referral to an inpatient specialty
Abraham et al., 2020	Citation searching	Wrong exposure	Barriers to transfer from the ED to the medical intensive care unit arose after the admission had been accepted
Adnan et al., 2021	Citation searching	Wrong exposure	Agreement between ED and discharge diagnoses; no dispute over admission
Alshyyab et al., 2023	Citation searching	Wrong exposure	Patient safety culture survey in two EDs; disputed admission referrals not reported separately
Aslaner et al., 2021	Citation searching	Wrong exposure	Before-and-after evaluation of consultation training; disputed acceptance was not the exposure
Buchman, 2021	Citation searching	Wrong exposure	Nurse handover of stroke patients from the ED to interventional radiology; no dispute over admission
Cheng et al., 2018	Citation searching	Wrong exposure	Factors associated with missed ED wait-time targets; contested responsibility not identified
Chiu et al., 2003	Citation searching	Wrong exposure	Agreement between ED admission and discharge diagnoses; no dispute over admission
Chladek et al., 2021	Citation searching	Wrong population	Paediatric ED only
Curtis et al., 2026	Citation searching	Wrong exposure	Trial of an emergency nursing framework for clinical handover during ED care
Daniels et al., 2018	Citation searching	Wrong exposure	Preventability of admissions judged after admission; no dispute over acceptance
Dúason et al., 2021	Citation searching	Wrong exposure	Handover from ambulance crews to ED staff
Freund et al., 2013	Citation searching	Wrong exposure	Medical errors in the ED; no dispute over admission
Gentili et al., 2021	Citation searching	Wrong population	Community cohort of older adults; not patients referred from the ED for admission
Gonzalo et al., 2014	Citation searching	Wrong exposure	Overnight adverse events on medical units; disputed acceptance not reported separately
Gu et al., 2012	Citation searching	Wrong exposure	Nurses’ views of handoffs in general; no dispute over admission
Güran et al., 2024	Citation searching	Wrong exposure	Effect of curfews on ED attendance and orthopaedic trauma
Guttmann et al., 2011	Citation searching	Wrong exposure	Waiting times and outcomes in patients who left the ED without admission
Habib and Sudaryo, 2023	Citation searching	Wrong exposure	ED length of stay and in-hospital mortality; disputes not measured
Hajzargarbashi et al., 2019	Citation searching	Wrong exposure	Waiting time for specialist consultation; refusal or redirection not shown
Hilligoss et al., 2015	Citation searching	Wrong publication type	Conceptual framework article drawing on the same ethnography as an included report [39]
Hodkinson, 2026	Citation searching	Wrong exposure	Development of emergency medicine in South Africa; disputed referrals not reported separately
Horwitz et al., 2009	Citation searching	Wrong exposure	Voicemail sign-out where ED physicians already admitted without inpatient approval
Kalyani et al., 2019	Citation searching	Wrong exposure	Handover from paramedics to ED staff
Kessler et al., 2012 (consultation)	Citation searching	Wrong exposure	Qualitative analysis of ED consultation problems; contested acceptance of admission not reported separately
Kessler et al., 2012 (training trial)	Citation searching	Wrong exposure	Consultation training with simulated consultants; no real admission referrals
Kirk et al., 2024	Citation searching	Wrong exposure	Specialty negotiation during simulation and planning of a new ED, not real admission referrals
Lee et al., 2017	Citation searching	Wrong exposure	Survey of ED-to-inpatient transition practices; contested acceptance not reported separately
Mamalelala et al., 2023	Citation searching	Wrong exposure	Nurses’ views of ED-to-intensive care handover; no refusal or dispute
Mathura et al., 2025	Citation searching	Wrong exposure	General medicine staffing in the ED and physician wellness; no dispute over admission
Meisel et al., 2015	Citation searching	Wrong exposure	Handover from emergency medical services to the ED
Momesso et al., 2023	Citation searching	Wrong exposure	Removal of the four-hour access standard
Murad et al., 2014	Citation searching	Wrong exposure	Information handoff from prehospital providers to the ED
Nikolla et al., 2026	Citation searching	Wrong exposure	ED physician turnover
Norman et al., 2018	Citation searching	Wrong exposure	Deterioration-risk screening before transfer from the ED
O’Connor et al., 2020	Citation searching	Wrong exposure	Nurse-to-nurse communication for patients already allocated to a ward
Peltonen et al., 2020	Citation searching	Wrong exposure	Reasons for ED transfer delays; contested acceptance not shown
Pittman et al., 2017	Citation searching	Wrong exposure	Emergency medicine graduates’ preparedness to manage fractures
Popovici et al., 2015	Citation searching	Wrong exposure	Technological problems during ED-to-medicine handover; no refusal or dispute
Pradhan et al., 2026	Citation searching	Wrong exposure	Factors associated with prolonged ED length of stay; disputes not identified
Rantala et al., 2021	Citation searching	Wrong exposure	Patients’ experience of boarding while waiting for a bed
Rathlev et al., 2014	Citation searching	Wrong exposure	Patient placement manager to reduce placement errors; disputed acceptance not studied
Robichaud, 2021	Citation searching	Wrong exposure	Handover from paramedics to ED staff (thesis)
Santos Gomes et al., 2026	Citation searching	Wrong exposure	Initiatives to reduce ambulance-to-ED handover delays
Schmitz and Strauss, 2023	Citation searching	Wrong publication type	Ethics essay on turfing
Scott et al., 2018	Citation searching	Wrong publication type	Perspective article on the four-hour rule
Singleton et al., 2018	Citation searching	Wrong exposure	Electronic sign-out for routine ED-to-medicine admissions; no disputed referrals
Stock et al., 2024	Citation searching	Wrong exposure	Physician assistant and nurse practitioner orthopaedic consultation service; no dispute over admission
Sullivan et al., 2016	Citation searching	Wrong exposure	Four-hour target compliance and inpatient mortality
Tarkie et al., 2023	Citation searching	Wrong exposure	Patterns of adult ED care and delay; contested acceptance not shown
Tasi et al., 2021	Citation searching	Wrong exposure	Handoff times to an ED observation unit after bed assignment
Taype-Huamaní et al., 2019	Citation searching	Not in English	Full text in Spanish
Taype-Huamaní et al., 2021	Citation searching	Not in English	Full text in Spanish
Thobaity, 2025	Citation searching	Wrong exposure	Bottlenecks in ED workflow
Traub et al., 2016	Citation searching	Wrong exposure	Physician-in-triage model within the ED
Wang et al., 2020	Citation searching	Wrong exposure	ED crowding and patient anxiety
Zhao et al., 2026	Citation searching	Wrong exposure	Triage decision-making of ED nurses
Zhiting et al., 2020	Citation searching	Wrong exposure	Validation of a triage scale

**Table 11:** PRISMA 2020 checklist PRISMA: Preferred Reporting Items for Systematic Reviews and Meta-Analyses Checklist items follow Page et al. [10]

Section	Item	Checklist item	Where reported
Title	1	Identify the report as a systematic review	Title
Abstract	2	Abstract	Abstract
Introduction	3	Rationale	Introduction and Background, paragraphs 1 to 3
Introduction	4	Objectives	Introduction and Background, paragraph 4
Methods	5	Eligibility criteria	Methods: Eligibility Criteria
Methods	6	Information sources	Methods: Information Sources and Search Strategy; Protocol and Registration (dates)
Methods	7	Search strategy	Appendices, Tables 5 to 8
Methods	8	Selection process	Methods: Study Selection
Methods	9	Data collection process	Methods: Data Extraction
Methods	10a	Outcomes sought	Methods: Eligibility Criteria
Methods	10b	Other variables sought	Methods: Data Extraction
Methods	11	Study risk of bias assessment	Methods: Risk of Bias and Quality Appraisal
Methods	12	Effect measures	Methods: Synthesis
Methods	13a	Eligibility for each synthesis	Methods: Synthesis
Methods	13b	Data preparation for synthesis	Methods: Synthesis
Methods	13c	Tabulation and display of results	Methods: Synthesis; Tables 1 to 4
Methods	13d	Methods of synthesis	Methods: Synthesis
Methods	13e	Exploring heterogeneity	Methods: Synthesis
Methods	13f	Sensitivity analyses	Methods: Synthesis (not performed)
Methods	14	Reporting bias assessment	Methods: Synthesis (not assessed, reason given)
Methods	15	Certainty assessment	Methods: Synthesis (GRADE-CERQual for qualitative findings)
Results	16a	Study selection	Results: Study Selection; Figure 1
Results	16b	Studies excluded at full text	Appendices, Table 10
Results	17	Study characteristics	Results: Study Characteristics; Table 1; Appendices, Table 9
Results	18	Risk of bias in studies	Results: Methodological Quality; Table 2
Results	19	Results of individual studies	Results: Measured Patient Outcomes; Table 3
Results	20a	Characteristics and risk of bias of contributing studies	Results: Study Characteristics; Methodological Quality
Results	20b	Results of syntheses	Results: Measured Patient Outcomes to Mitigating Strategies; Tables 3 and 4
Results	20c	Investigations of heterogeneity	Not applicable (no statistical synthesis)
Results	20d	Sensitivity analyses	Not performed
Results	21	Reporting biases	Not assessed (Methods: Synthesis)
Results	22	Certainty of evidence	Table 4; Discussion: Principal Findings
Discussion	23a	Interpretation in context of other evidence	Discussion: Principal Findings; Comparison With Previous Work
Discussion	23b	Limitations of the evidence	Discussion: Strengths and Limitations
Discussion	23c	Limitations of the review processes	Discussion: Strengths and Limitations
Discussion	23d	Implications for practice, policy and research	Discussion: Implications for Practice; Implications for Research
Other information	24a	Registration	Methods: Protocol and Registration
Other information	24b	Protocol access	Methods: Protocol and Registration (PROSPERO CRD420261538117)
Other information	24c	Amendments to the protocol	Methods: Deviations From the Protocol
Other information	25	Support	Disclosures
Other information	26	Competing interests	Disclosures
Other information	27	Availability of data, code and other materials	Methods: Data Availability
